# Supplementary material for: Deciphering the catalytic mechanism of superoxide dismutase activity of carbon dot nanozyme
Source: Nat Commun. 2023 Jan 11;14:160. doi: 10.1038/s41467-023-35828-2 (PMC9834297; doi:10.1038/s41467-023-35828-2)
Supplement: Supplementary file 1 — Supplementary Information [file 41467_2023_35828_MOESM1_ESM.pdf]

Supplementary Information

Deciphering the catalytic mechanism of superoxide dismutase activity of carbon dot nanozyme

Wenhui Gao[1#], Jiuyang He[2#], Lei Chen[2,3#], Xiangqin Meng[2], Yana Ma[1], Liangliang Cheng[1] Kangsheng Tu[4], Xingfa Gao[5], Cui Liu[1*], Mingzhen Zhang[1,4*], Kelong Fan[2,6,7*], Dai-Wen Pang[8*] & Xiyun Yan[2,6,7*]

[1] *School of Basic Medical Sciences, Xi'an Jiaotong University Health Science Center, Xi'an, Shaanxi, 710061, P. R. China*

[2] *CAS Engineering Laboratory for Nanozyme, Key Laboratory of Protein and Peptide Pharmaceutical, Institute of Biophysics, Chinese Academy of Sciences, Beijing 100101, P. R.China*

[3] *Laboratory of Theoretical and Computational Chemistry, Institute of Theoretical Chemistry, Jilin University, Changchun 130023, P. R. China*

[4] *Department of Hepatobiliary Surgery, the First Affiliated Hospital of Xi'an Jiaotong University, Xi'an, Shaanxi, 710061, P. R. China*

[5] *National Center for Nanoscience and Technology, 100190, P. R. China.*

[6] *Nanozyme Medical Center, School of Basic Medical Sciences, Zhengzhou University, Zhengzhou 450052, P. R. China.*

[7] *University of Chinese Academy of Sciences, Beijing 101408, P. R. China.*

[8] *Haihe Laboratory of Sustainable Chemical Transformations, Tianjin Key Laboratory of Biosensing and Molecular Recognition, Frontiers Science Center for New Organic Matter, Research Center for Analytical Sciences, College of Chemistry, and Frontiers Science Center for Cell Responses, Nankai University, Tianjin 300071, P. R. China.*

[*]Corresponding authors: liucui@xjtu.edu.cn (C. Liu), mzhang21@xjtu.edu.cn (M. Zhang), fankelong@ibp.ac.cn (K. Fan), dwpang@whu.edu.cn (D.-W. Pang), and yanxy@ibp.ac.cn (X. Yan).

[#] These authors contributed equally to this work.

Supplementary Methods 3
Supplementary Figures 6
Supplementary Tables.. 14
Supplementary References.. 16

Supplementary Methods

Synthesis

Synthesis of C-dots-HCl: C-dots (5 mg) was added to HCl solution (0.1 M, 10 mL) and then refluxed for 12 h. The resulting solution was neutralized with $NaHCO_3$ and then dialyzed for 3 d.

Synthesis of C-dots-Cy5.5: 5 mg of C-dots and 2-3 mL of thionyl chloride ($SOCl_2$) were added to 6-8 mL of acetonitrile. The mixture was heated at 80~100 °C until all of C-dots solid powder dispersed. The C-dots acyl chloride (solid) was obtained and redispersed in 6-8 mL of anhydrous acetonitrile after removing excess $SOCl_2$ and acetonitrile by distillation. Then, 167 μL of Sulfo-Cyanine5.5 amine (3 mg/mL in DMF) was added to the solution of C-dots acyl chloride. After continue stirring for 30 min at room temperature in dark, the solvent was removed by rotary evaporation. The resulting product was redispersed in water and purified by agarose gel electrophoresis (1%).

The CAT-like activity of C-dots

The CAT-like activity of C-dots was detected by monitoring the elimination of H_2O_2 and generation of oxygen. The concentration of H_2O_2 was monitored in time-drive mode at 240 nm by using a UV-Vis spectrophotometer. The reaction solutions contained 10 mM H_2O_2 and C-dots with a concentration of 10 μg/mL in 500 μL of PBS (25 mM PBS, pH 7.4). The absorbance peak at 240 nm of the solution was monitored by using a UV-vis spectroscopy. The oxygen generation was measured by using a specific oxygen electrode on Multi-Parameter Analyzer (JPSJ-605F, Leica China). In a typical test, 60 μL of 30% H_2O_2 solution was added to 14.94 mL of water, and then C-dots (final concentration of 10 μg/mL) was added. The generated oxygen solubility (unit: mg/L) was recorded from 0 to 60 s.

The POD/OXD-like activity of C-dots

The POD-like activity assays of C-dots (10 μg/mL) were carried out using TMB (1 mM) as the substrate in the presence of 1 M H_2O_2 in citric acid-NaAc buffer solution. The absorbance (at 652 nm for ox-TMB) of the reaction was recorded using a UV-vis spectroscopy. The OXD-like activity of C-dots were tested under the same condition in the absence of H_2O_2.

Measurement of ESR

The free radicals were quantitatively estimated by the ESR signal intensity of the free radical spin adduct using the peak-to-peak height of the line of the ESR spectrum. Superoxide radicals were produced by 13 mM L-met and 20 μM riboflavin (rib) in 25 mM PBS buffer under LED irradiation of 5 min. DMPO (25 mM) was used to trap $O_2^{\bullet-}$ and generate the spin adduct (BMPO/$^{\bullet}$OOH). ESR spectra were recorded in the absence and presence of C-dots (5, 10, 20, 30, 40, and 50 μg/mL) at 4.97 mW microwave power, 6 G modulation amplitude, and 200 G scan range.

Theoretical calculation

All geometries, including local minima and transition states (TS), were fully optimized by employing the B3LYP density functional[1] and the 6-31G(d,p) basis set[2]. In order to identify whether the stationary point is a local minimum or a transition state and obtain the Gibbs free energy, the harmonic frequency analysis was calculated for each structure. In our calculations, the PCM solvation model was used to model the water environment. All the calculations were carried out using the Gaussian 09 package (Gaussian, Inc. Wallingford CT, USA, 2009).

Intracellular ROS/$O_2^{\bullet-}$ scavenging in RAW264.7 cells

RAW264.7 cell line was purchased form Pricella, CL-1090. The fluorescent probe 2',7'-dichlorofluorescein diacetate (DCFH-DA) and dihydroethidium (DHE) were used to measure the generation of intracellular ROS and $O_2^{\bullet-}$, respectively. Briefly, RAW264.7 cells were first seeded in a 6-well plate and cultured for 12 h. After

that, the cell medium was removed, and the adherent cells were incubated with C-dots (100 μg/mL) for 8 h. To remove the excess nanoparticles, cells were washed three times with PBS, and the cells were incubated for another 1 h with 1 mL of PBS containing the Rosup (0.5 mg/mL). Then, RAW264.7 cells were further incubated with 1 mL of PBS containing DCFH-DA (10 μM) or DHE (10 μM) for 45 min. Finally, the cells were washed three times with PBS. Cell images were observed using a fluorescent microscope.

Supplementary Figures

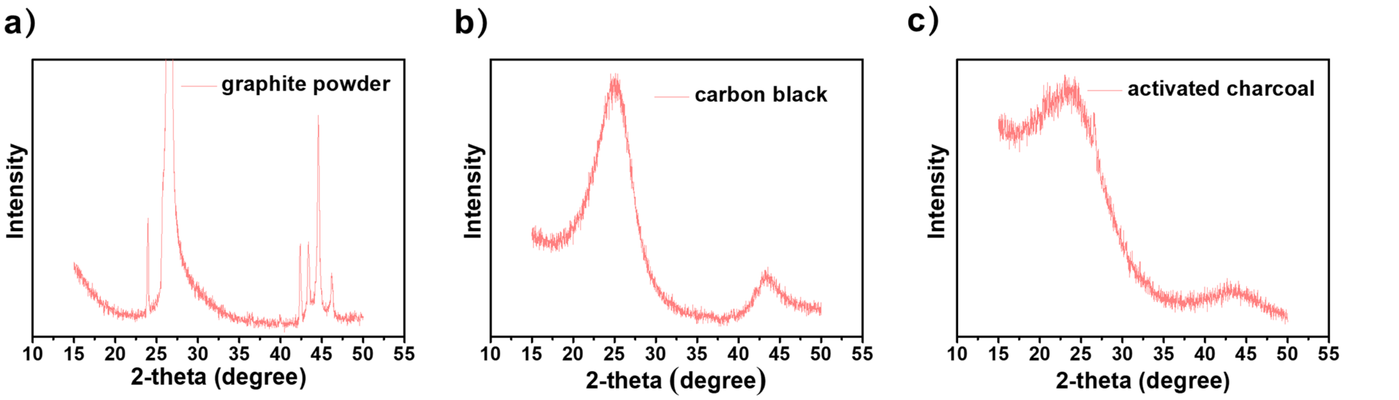

Supplementary Figure 1 XRD patterns of raw carbon materials for preparing C-dots. (**a**) graphite powder, (**b**) carbon black, (**c**) activated charcoal.

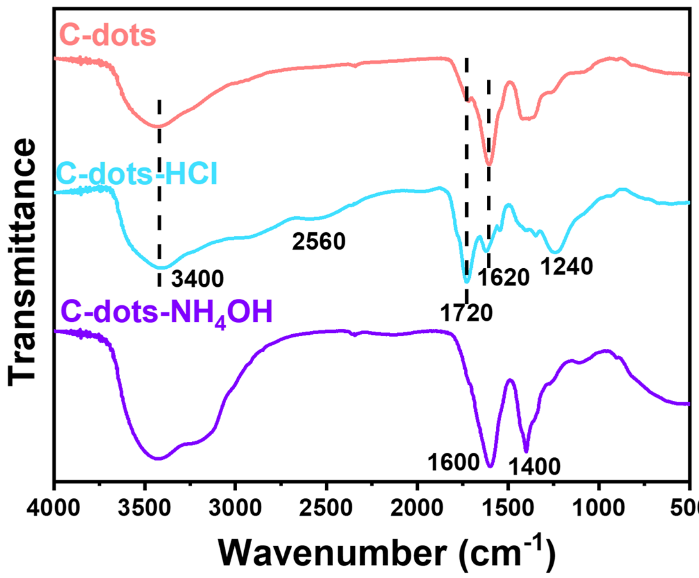

Supplementary Figure 2 FT-IR spectra of C-dots (untreated), C-dots-HCl (acidified), and C-dots-NH_4OH (alkalified).

In this work, we performed a series of chemical modifications to transform the functional groups, especially hydroxyl and carboxyl groups, which involved changes in FT-IR. In order to accurately identify the absorption O-H, we carried out strict drying treatment on the samples, so that the O-H on the surface of the C-dots can be determined by the absorption band around 3400 cm^{-1}. The broadband around 2560 cm^{-1} could be attributed

to the stretching vibration of the hydrogen bond of associating carboxyl group, which enabled one to distinguish a carboxylic acid from all the other carbonyl compounds[3]. For verifying the existence of carboxyl groups on the surface of C-dots, the sample was acidified or alkalified by HCl or NH_4OH before FT-IR measurement. As shown in Supplementary Figure 2, the peaks at 3400, 2560, 1720, and 1240 cm^{-1} in the FT-IR spectrum of C-dots-HCl increased significantly. After reacted with NH_4OH, the peaks at 2560, 1720, and 1240 cm^{-1} disappeared. Two strong peaks at 1600 and 1400 cm^{-1} appeared, which according to asymmetric and symmetric stretching vibrations of −COO^-, respectively, because the carboxyl group (−COOH) was converted to carboxylate anion (−COO^-). Therefore, we can attribute the peaks at 1720, 1240, and 2560 cm^{-1} to the C=O, C−O, and associated hydrogen bond, respectively, of carboxylic acid. While, the peak at 1620 cm^{-1} is more likely to be attributed to the acid-insensitive C=C.

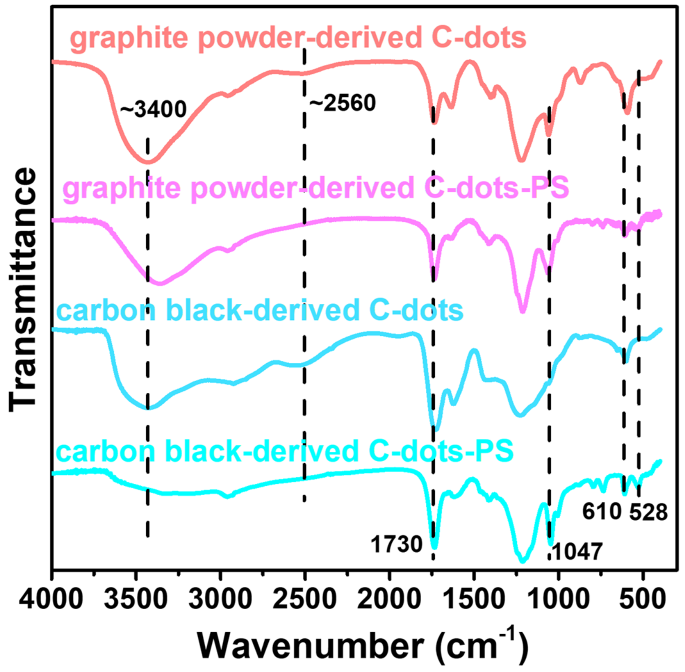

Supplementary Figure 3 FT-IR spectra of C-dots prepared from graphite powder, carbon black and their derivatives.

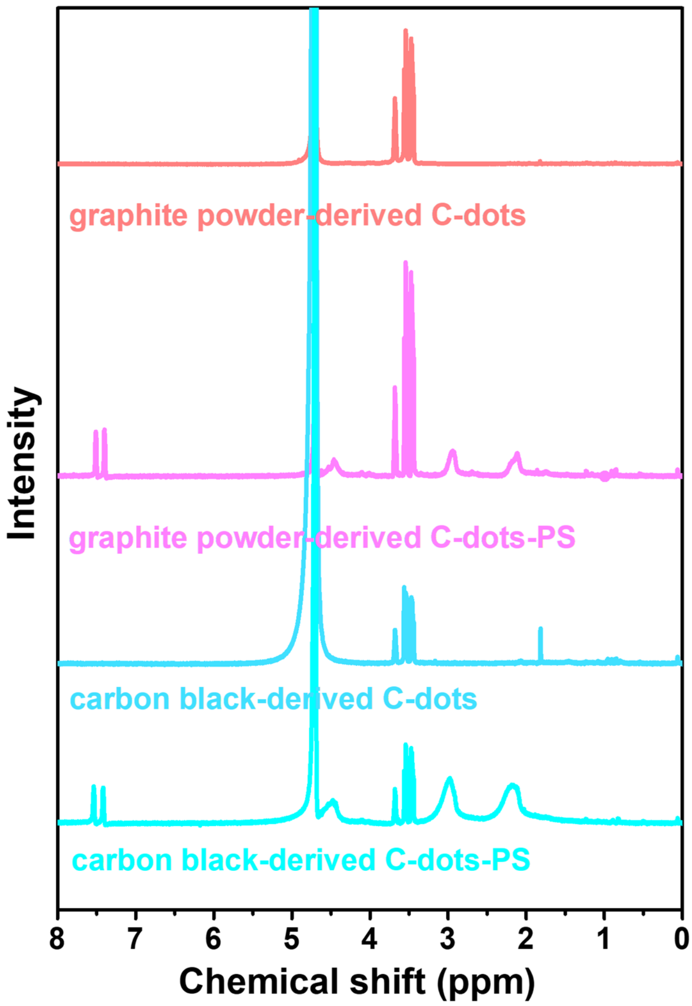

Supplementary Figure 4 ^{1}H NMR spectra of C-dots prepared from graphite powder, carbon black and their derivatives.

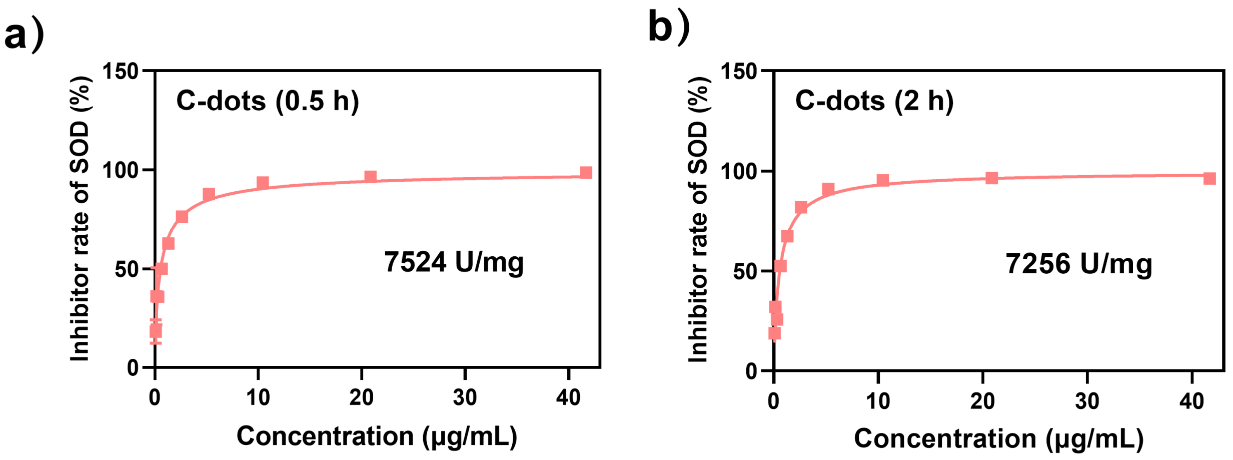

Supplementary Figure 5 The SOD-like activities of C-dots with reaction times of 0.5 (**a**) and 2 h (**b**). Data are presented as means ± SD from three independent experiments.

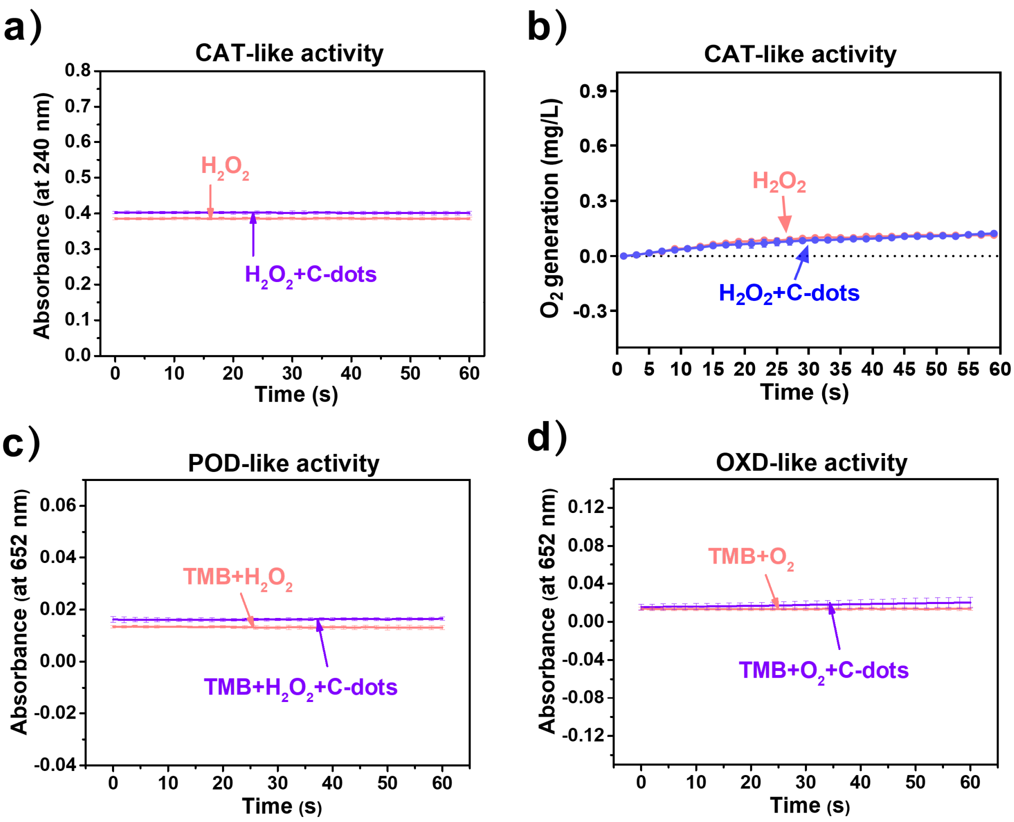

Supplementary Figure 6 Detection of CAT-like activity of C-dots by monitoring the elimination of H_2O_2 (**a**), and the generation of oxygen (**b**), detection of POD (**c**), and OXD-like (**d**) activities of C-dots. Data are presented as means ± SD from three independent experiments.

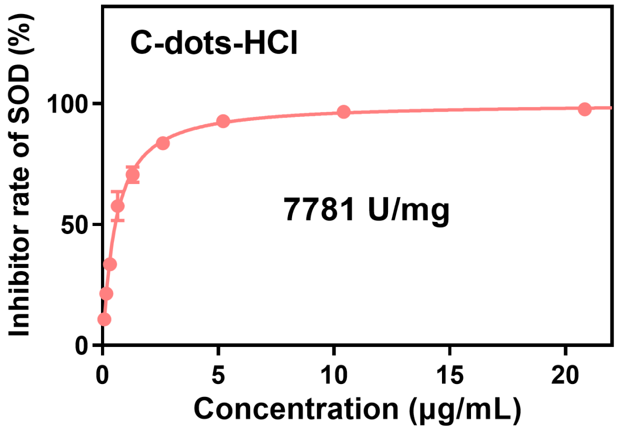

Supplementary Figure 7 The SOD-like activity of C-dots-HCl. Data are presented as means ± SD from three independent experiments.

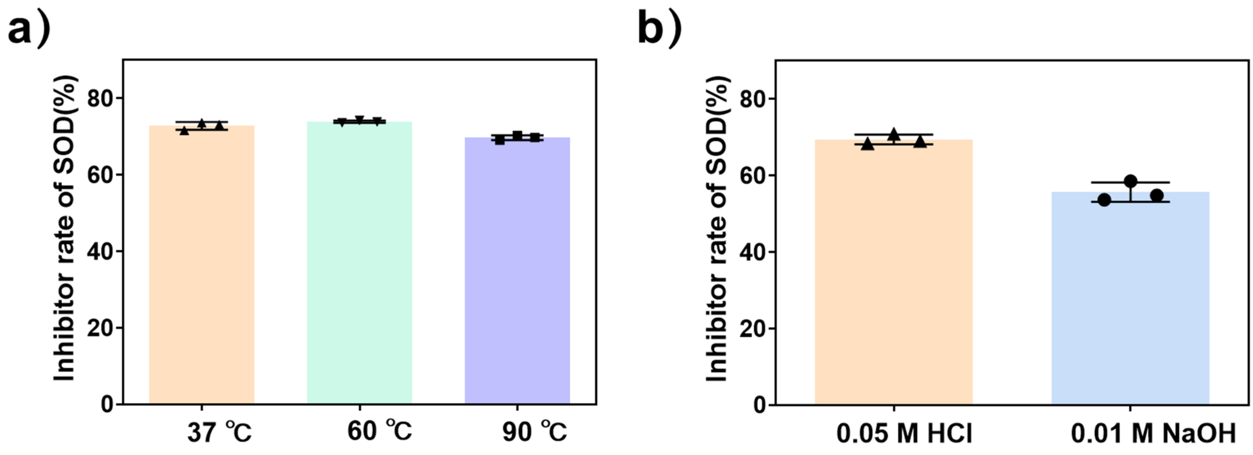

Supplementary Figure 8 The SOD-like activity change of C-dot nanozyme after different pretreatment. (**a**) The SOD-like activities of C-dot nanozyme after being treated in 37, 60, and 90 °C water bath for 1 h. (**b**) The SOD-like activities of C-dot nanozyme after pretreatment in 0.05 M HCl and 0.01 M NaOH solutions for 1 h. The SOD-like activity of C-dot nanozyme was tested by a total superoxide dismutase assay kit with WST-8 with a concentration of 50 μg/mL (S0101M, Beyotime). Data are presented as means ± SD from three independent experiments.

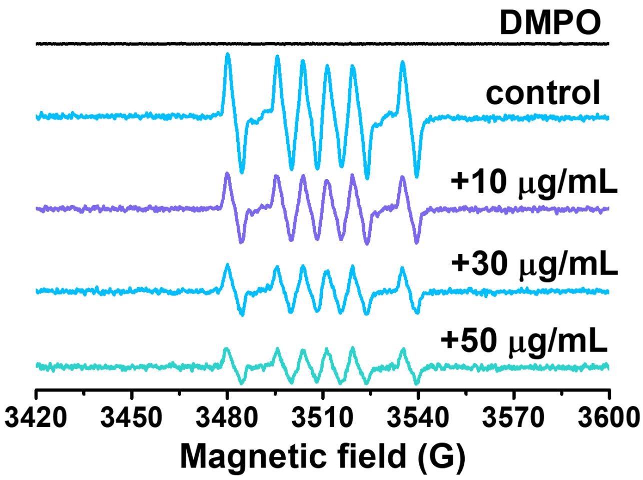

Supplementary Figure 9 ESR spectra of the mixed solution of L-methionine, riboflavin, and DMPO in the absence (control) and presence of C-dot SOD nanozyme with different concentrations (10~50 μg/mL).

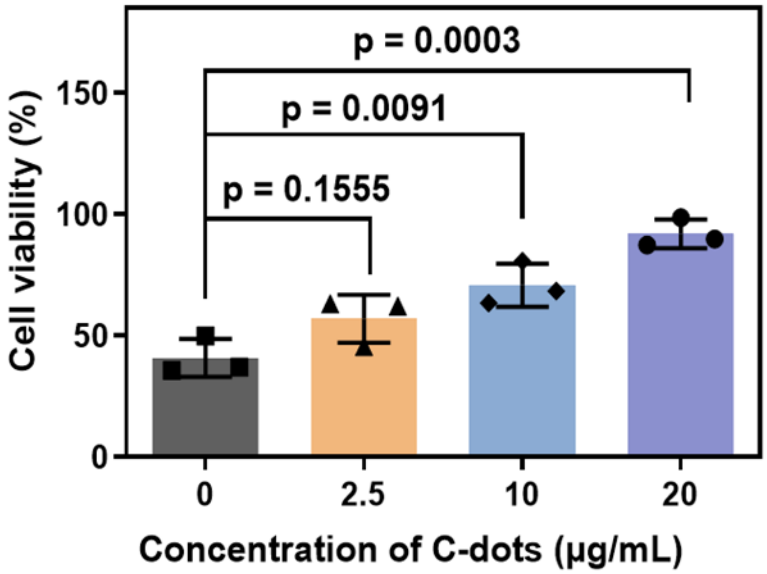

Supplementary Figure 10 Cell viabilities of SH-SY5Y co-incubated with C-dot SOD nanozyme (concentration ranging from 0 to 20 μg/mL) and paraquat (final concentration of 250 μM). Data are presented as means ± SD from three independent experiments. P values are determined with one-way ANOVA Tukey's multiple comparisons test.

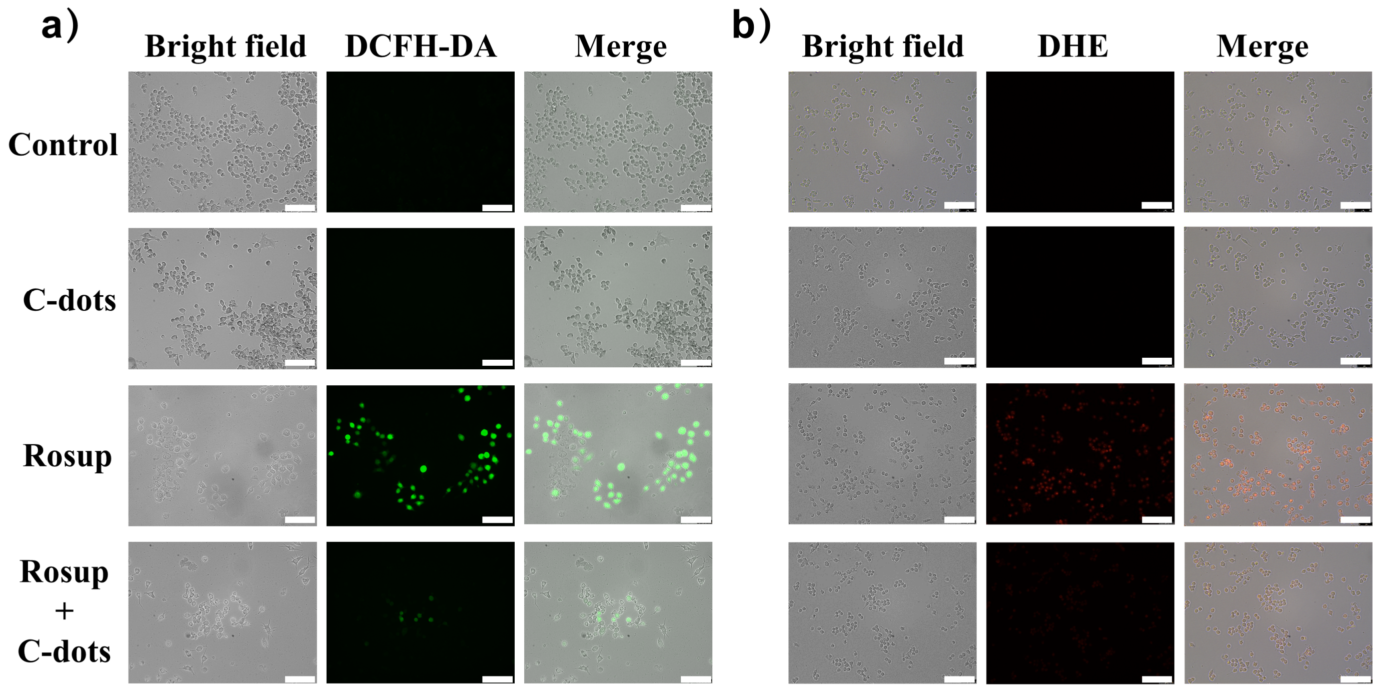

Supplementary Figure 11 *In vitro* ROS scavenging capacity of C-dot SOD nanozymes. (**a**) ROS level was detected by fluorescence of DCFH-DA in RAW264.7 macrophage cells under different conditions. (**b**) $O_2^{\bullet-}$ production detected by fluorescence of DHE in RAW264.7 macrophage cells under different conditions. n = 3 independent experiments. The scale bar is 100 μm.

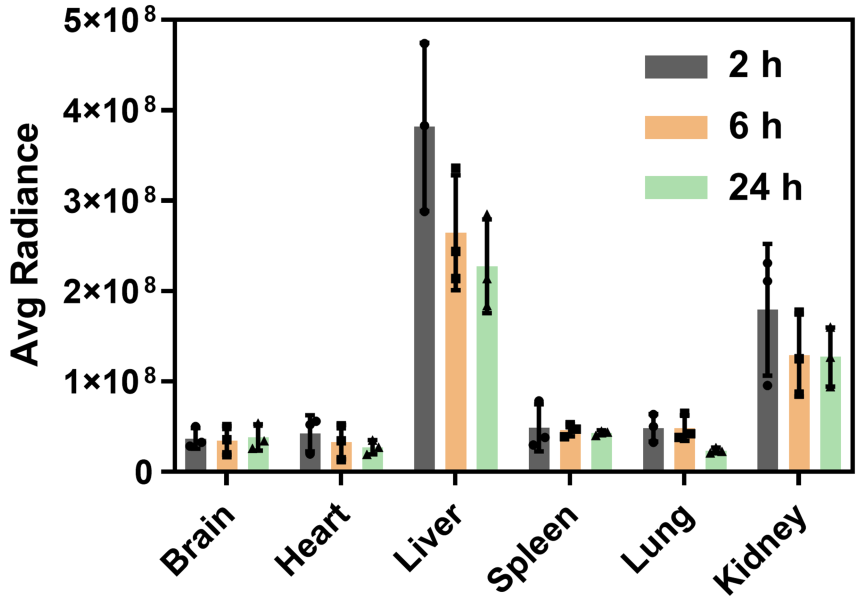

Supplementary Figure 12 The distribution of C-dot SOD nanozymes in major organs of ischemic stroke mice. Data are presented as means ± SD from three mice.

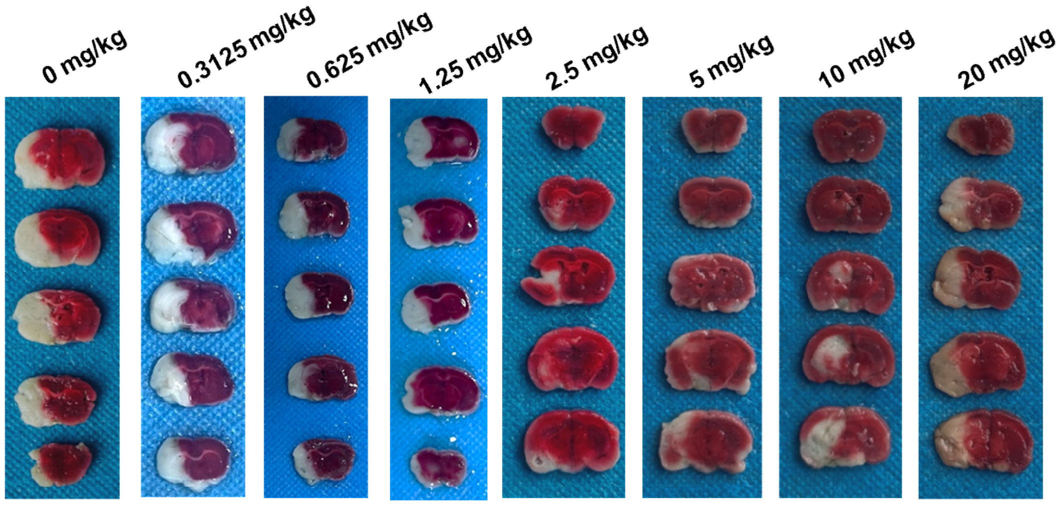

Supplementary Figure 13 Representative TTC-stained brain sections of C-dot SOD nanozyme dosages for the treatment of ischemic stroke model. n = 3 mice.

Supplementary Tables

Supplementary Table 1 Comparison of the typical parameters of SOD-like activities of C-dot nanozyme with the reported SOD nanozymes and natural SOD.

Nanozyme	[E] / μg•mL^{-1}	Assay kit	Specific activity / U·mg^{-1}	Inhibition rate / %	Ref.
**Pt NPs-PVP**	20	WST-1 by Sigma-Aldrich	/	58.30	4
**MFC-MSNs**	300	WST-1 by Sigma-Aldrich	/	~80	5
**$CeVO_4$**	40	WST-1 by Sigma-Aldrich	/	~100	6
**CaPB**	960	WST-8 by Beyotime	/	~100	7
**Rh-PEG NDs**	80	WST-8 by Beyotime	/	~93	8
**N-PCNSs**	400	WST-1 by Dojindo	/	~75	9
**PtPB**	20	WST-1 by Dojindo	/	~45	10
**CeO_2**	20	WST-1 by Dojindo	/	~60	11
**PB**	50	WST-1 by Dojindo	/	~90	12
**Fe_3O_4 NPs**	2000	WST-1 by Dojindo	5.65	~11	13
**Cu-SAzyme**	12.3	WST-1 by Dojindo	448.22	~61	14
**$MnPS_3$**	5.0	WST-1 by Dojindo	721.21	~70	15
**pero-nanozysome**	8.3	WST-1 by Dojindo	1257	~73	16
**natural SOD**	3.03	WST-1 by Dojindo	4743.8	~79	This work
**C-dot SOD Nanozyme**	2.60	WST-1 by Dojindo	10767	~87	

[E]: The nanozyme concentration.

Supplementary Table 2 Comparison of the oxygen-containing functional groups and specific activity of different C-dots (calculated by integrating fitting curve area of *C 1s* XPS).

Nanozyme	C=C	C-O	C=O	-COO	Specific activity (U/mg)
**graphite powder-derived C-dots**	57.2%	27.8%	4.2%	10.8%	405
**carbon black-derived C-dots**	63.9%	14.1%	6.8%	15.2%	418
**C-dots (0.5 h)**	71.9%	9.8%	7.3%	11.0%	7524
**C-dots (1.5 h)**	70.7%	8.6%	9.2%	11.5%	10767
**C-dots (2 h)**	73.2%	9.1%	7.4%	10.3%	7256
**C-dots-$NaBH_4$**	68.9%	15.6%	5.3%	10.2%	1060
**C-dots-$NaBH_4$-HNO_3**	63.3%	14.5%	13.3%	8.9%	3845
**C-dots-NaOH-200 °C**	73.9%	10.0%	3.7%	12.4%	810
**C-dots-NaOH-HI**	79.8%	11.6%	0.00%	8.6%	100
**C-dots-PS**	70.7% [a]	passivation	9.2% [a]	passivation	1563
**C-dots-PS-HCl**	70.7% [a]	passivation	9.2% [a]	11.5% [a]	4043
**C-dots-NaOH-40 °C**	69.8%	12.2%	4.4%	13.6%	448

[a] *Only the contents of hydroxyl and carboxyl groups on the surface of C-dots-PS are passivated, and the contents of other groups remain unchanged.*

Supplementary References

1. Becke, A. D. Density-functional thermochemistry. III. The role of exact exchange. *J. Chem. Phys.* **98,** 5648-5652 (1993).

2. Petersson, G. A. & Allaham, M. A. A complete basis set model chemistry. II. Open-shell systems and the total energies of the first-row atoms. *J. Chem. Phys.* **94,** 6081-6090 (1991).

3. Furniss, B. S., Hannaford, A. J., Smith, P. W. & Tatchell, A. R. Vogel's textbook of practical organic chemistry (Pearson Prentice Hall. London, 1989).

4. Zhang, D.-Y. et al. Ultrasmall platinum nanozymes as broad-spectrum antioxidants for theranostic application in acute kidney injury. *Chem. Eng. J.* **409,** 127371 (2021).

5. Kim, J. et al. Synergistic oxygen generation and reactive oxygen species scavenging by manganese ferrite/ceria co-decorated nanoparticles for rheumatoid arthritis treatment. *ACS Nano* **13,** 3206-3217 (2019).

6. Singh, N., NaveenKumar, S.K., Geethika, M. & Mugesh, G. A cerium vanadate nanozyme with specific superoxide dismutase activity regulates mitochondrial function and ATP synthesis in neuronal cells. *Angew. Chem. Int. Edit.* **60,** 3121-3130 (2021).

7. Wang, K. et al. Engineering ultrasmall ferroptosis-targeting and reactive oxygen/nitrogen species-scavenging nanozyme for alleviating acute kidney injury. *Adv. Funct. Mater.* **32,** 2109221 (2022).

8. Miao, Z. et al. Ultrasmall rhodium nanozyme with RONS scavenging and photothermal activities for anti-inflammation and antitumor theranostics of colon diseases. *Nano Lett.* **20,** 3079-3089 (2020).

9. Fan, K. et al. *In vivo* guiding nitrogen-doped carbon nanozyme for tumor catalytic therapy. *Nat. Commun.* **9,** 1440 (2018).

10. Li, Z.-H., Chen, Y., Sun, Y. & Zhang, X.-Z. Platinum-doped prussian blue nanozymes for multiwavelength bioimaging guided photothermal therapy of tumor and anti-inflammation. *ACS Nano* **15,** 5189-5200 (2021).

11. Zhao, S. et al. An orally administered CeO_2@Montmorillonite nanozyme targets inflammation for inflammatory bowel disease therapy. *Adv. Funct. Mater.* **30,** 2004692 (2020).

12. Sahu, A., Jeon, J., Lee, M.S., Yang, H.S. & Tae, G. Antioxidant and anti-inflammatory activities of Prussian blue nanozyme promotes full-thickness skin wound healing. *Mater. Sci. Eng. C-Mater. Biol. Appl.* **119,** 111596 (2021).

13. Yan, B. et al. Dietary Fe_3O_4 nanozymes prevent the injury of neurons and blood-brain barrier integrity from cerebral ischemic stroke. *ACS Biomater. Sci. Eng.* **7,** 299-310 (2021).

14. Yang, J. et al. Bioinspired copper single-atom nanozyme as a superoxide dismutase-like antioxidant for sepsis treatment. *Exploration.* **2,** 20210267 (2022).

15. Zhang, C. et al. Machine learning guided discovery of superoxide dismutase nanozymes for androgenetic alopecia. *Nano Lett.* **22,** 8592–8600 (2022).

16. Xi, J. et al. A nanozyme-based artificial peroxisome ameliorates hyperuricemia and ischemic stroke. *Adv. Funct. Mater.* **31,** 2007130 (2021).
