# Supplementary material for: Deciphering the catalytic mechanism of superoxide dismutase activity of carbon dot nanozyme
Source: Nat Commun. 2023 Jan 11;14:160. doi: 10.1038/s41467-023-35828-2 (PMC9834297; doi:10.1038/s41467-023-35828-2)
Supplement: Supplementary file 3 — Reporting Summary [file 41467_2023_35828_MOESM3_ESM.pdf]

nature portfolio

Corresponding author(s): Kelong Fan

Last updated by author(s): Dec 24, 2022

Reporting Summary

Nature Portfolio wishes to improve the reproducibility of the work that we publish. This form provides structure for consistency and transparency in reporting. For further information on Nature Portfolio policies, see our Editorial Policies and the Editorial Policy Checklist.

Statistics

For all statistical analyses, confirm that the following items are present in the figure legend, table legend, main text, or Methods section.

n/a	Confirmed	
☐	☒	The exact sample size (n) for each experimental group/condition, given as a discrete number and unit of measurement
☐	☒	A statement on whether measurements were taken from distinct samples or whether the same sample was measured repeatedly
☐	☒	The statistical test(s) used AND whether they are one- or two-sided<br>*Only common tests should be described solely by name; describe more complex techniques in the Methods section.*
☒	☐	A description of all covariates tested
☐	☒	A description of any assumptions or corrections, such as tests of normality and adjustment for multiple comparisons
☐	☒	A full description of the statistical parameters including central tendency (e.g. means) or other basic estimates (e.g. regression coefficient) AND variation (e.g. standard deviation) or associated estimates of uncertainty (e.g. confidence intervals)
☐	☒	For null hypothesis testing, the test statistic (e.g. F, t, r) with confidence intervals, effect sizes, degrees of freedom and P value noted<br>*Give P values as exact values whenever suitable.*
☒	☐	For Bayesian analysis, information on the choice of priors and Markov chain Monte Carlo settings
☒	☐	For hierarchical and complex designs, identification of the appropriate level for tests and full reporting of outcomes
☐	☒	Estimates of effect sizes (e.g. Cohen's d, Pearson's r), indicating how they were calculated

Our web collection on statistics for biologists contains articles on many of the points above.

Software and code

Policy information about availability of computer code

Data collection	Transmission electron microscopy (TEM) images were obtained by using a FEI Tecnai G2 F30 (FEI, USA) at an acceleration voltage of 300 kV. Powder X-ray diffraction (XRD) data were collected by using a Bruker D8 ADVANCE (Germany) with a scan rate of 6 ° / min. FT-IR spectra were recorded by a Thermo Fisher Nicolet 5700 (USA). Raman spectra were performed by a Thermo Fisher DXR2xi Raman Imaging Microscope (USA) under excitation wavelength of 532 nm. The proton magnetic resonance (1H NMR) spectra were recorded using an AVANCE III HD (USA) spectrometer (600 MHz, D2O as solvent). X-ray photoelectron spectroscopy (XPS) spectra were recorded by a Thermo Escalab 250Xi (USA). Electron spin resonance (ESR) spectra were recorded by a Bruker A300-9.5/12 (Switzerland) at room temperature. Confocal laser scanning microscopy images were obtained by Olympus FluoView FV-1000 (Japan). Flow cytometry data were collected by FACS CaliburTM, Becton Dickinson (USA). Microplate absorbance was measured using Tecan Spark 20M multi-mode microplate reader, Switzerland. The fluorescence imaging was performed on an in vivo imaging instrument (IVIS Lumina 3, PE, USA), and images were prepared by IVIS Living Image 3.0 software (PerkinElmer, USA). Tissue sections were imaged by the Leica DM3000 microscope (Leica, Wetzlar, Germany). All the calculations were carried out using the Gaussian 09 package (Gaussian, Inc., Wallingford CT).
Data analysis	General statistical data were analyzed by Image J (1.53t), Origin 8, Nano Measurer 1.2, MestReNova 5.3.1-4696, XPSPEAK41, DigitalMicrograph 3.7.4, ZEN 2010, FlowJo 7.6.1 and Graphpad prism 8.

For manuscripts utilizing custom algorithms or software that are central to the research but not yet described in published literature, software must be made available to editors and reviewers. We strongly encourage code deposition in a community repository (e.g. GitHub). See the Nature Portfolio guidelines for submitting code & software for further information.

Data

Policy information about availability of data

All manuscripts must include a data availability statement. This statement should provide the following information, where applicable:

- Accession codes, unique identifiers, or web links for publicly available datasets
- A description of any restrictions on data availability
- For clinical datasets or third party data, please ensure that the statement adheres to our policy

Data supporting the findings of this work are available within the paper and its Supplementary Information files. Source data are provided with this paper and the raw data are available upon request to the corresponding authors.

Human research participants

Policy information about studies involving human research participants and Sex and Gender in Research.

Reporting on sex and gender	N/A
Population characteristics	N/A
Recruitment	N/A
Ethics oversight	N/A

Note that full information on the approval of the study protocol must also be provided in the manuscript.

Field-specific reporting

Please select the one below that is the best fit for your research. If you are not sure, read the appropriate sections before making your selection.

[x] Life sciences [ ] Behavioural & social sciences [ ] Ecological, evolutionary & environmental sciences

For a reference copy of the document with all sections, see nature.com/documents/nr-reporting-summary-flat.pdf

Life sciences study design

All studies must disclose on these points even when the disclosure is negative.

Sample size	Sample size choice in the manuscript was consistent with previous studies (ref. He, X., Zhang, L., Queme, L. et al, 2018. https://doi.org/10.1038/nm.4483; Li, F., Sun, H., Ren, J. et al, 2022. https://doi.org/10.1038/s41467-022-35022-w). Exact sample size for each experiment is shown in the figure legend.
Data exclusions	No data was excluded from the analysis.
Replication	Independent replicates of at least 3 times were used for all experiments. We confirmed that the attempts at replication were successful. Each figure contains detailed independent experimental replicates in the figure.
Randomization	All samples/organisms were randomly allocated into experimental groups.
Blinding	Blinding is not applicable for the synthesis and characterization of nanomaterials. For in vivo experiments, all the investigators were blinded to group allocation during data collection and analysis.

Reporting for specific materials, systems and methods

We require information from authors about some types of materials, experimental systems and methods used in many studies. Here, indicate whether each material, system or method listed is relevant to your study. If you are not sure if a list item applies to your research, read the appropriate section before selecting a response.

Materials & experimental systems

n/a	Involved in the study
[ ]	[x] Antibodies
[ ]	[x] Eukaryotic cell lines
[x]	[ ] Palaeontology and archaeology
[ ]	[x] Animals and other organisms
[x]	[ ] Clinical data
[x]	[ ] Dual use research of concern

Methods

n/a	Involved in the study
[x]	[ ] ChIP-seq
[ ]	[x] Flow cytometry
[x]	[ ] MRI-based neuroimaging

Antibodies

Antibodies used: Anti-LAMP1 antibody [EPR21026] ab208943 was used in this study.

Validation: https://www.abcam.com/lamp1-antibody-epr21026-ab208943.html

Eukaryotic cell lines

Policy information about cell lines and Sex and Gender in Research

Cell line source(s): SH-SY5Y cell line and RAW 264,7 cell line were purchased form Pricella.

Authentication: A short tandem repeat DNA profiling method was used to authenticate the cell lines and the results were compared withreference database.

Mycoplasma contamination: The cell lines were tested as mycoplasma negative by a standard PCR protocols.

Commonly misidentified lines (See ICLAC register): No commonly misidentified cell lines were used in this study.

Animals and other research organisms

Policy information about studies involving animals; ARRIVE guidelines recommended for reporting animal research, and Sex and Gender in Research

Laboratory animals: C57BL/6J mice, male, 8-10 weeks. All mice were group-housed 5 mice per cage in temperature (22-26°C) and humidity (40%-70%) housing rooms on a 12h light, 12h dark cycle.

Wild animals: The study did not involve wild animals.

Reporting on sex: The study only used male C57BL/6J mice, because based on previous studies, female mice present minor brain infarct in the acute phase of ischemic stroke relative to male caused by differences in hormone, genomic factors, vascular anatomy and immune response. (ref. Jiang, Ming, et al. 2020. https://doi.org/10.3389/fneur.2020.00504)

Field-collected samples: This study did not involve samples collected from the field.

Ethics oversight: The ethics oversight is subject to the approval and guidance of the Institutional Animal Care and Use Committee of the Institute of Biophysics, Chinese Academy of Sciences.

Note that full information on the approval of the study protocol must also be provided in the manuscript.

Flow Cytometry

Plots

Confirm that:

- [x] The axis labels state the marker and fluorochrome used (e.g. CD4-FITC).
- [x] The axis scales are clearly visible. Include numbers along axes only for bottom left plot of group (a 'group' is an analysis of identical markers).
- [x] All plots are contour plots with outliers or pseudocolor plots.
- [x] A numerical value for number of cells or percentage (with statistics) is provided.

Methodology

Sample preparation: For intracellular accumulation of C-dots-Cy5.5 in SH-SY5Y cells, cells were plated at 150000 cells / well in six-well plates and allowed to settle overnight for adherence. C-dots-Cy5.5 were then added into wells for 8 h of incubation in the presence of

	H_2O_2. The fluorescence intensities were examined using flow cytometry. For ROS/O_2•– level detection in SH-SY5Y cells, cells were plated in six-well plates and allowed to settle overnight for adherence. C-dots were then added into wells for 24 h of incubation in the presence of paraquat. Then, SH-SY5Y cells were further incubated with 1 mL of PBS containing DCFH-DA or DHE for 45 min. Finally, the fluorescence intensities were examined using flow cytometry.
Instrument	FACS CaliburTM, Becton Dickinson, Franklin Lakes, NJ, USA
Software	FlowJo 7.6 software
Cell population abundance	10^8-10^9 cells/mL analyzed by cellometer auto t4
Gating strategy	Cells were gated based on size and granularity of forward and side scatter (SSC and FCS) and the cell gate is analyzed for specific fluorescence.

☒ Tick this box to confirm that a figure exemplifying the gating strategy is provided in the Supplementary Information.
